# Inflammation in Heart Failure—Future Perspectives

**DOI:** 10.3390/jcm12247738

**Published:** 2023-12-17

**Authors:** Alexandru Mircea Arvunescu, Ruxandra Florentina Ionescu, Sanda Maria Cretoiu, Silviu Ionel Dumitrescu, Ondin Zaharia, Ioan Tiberiu Nanea

**Affiliations:** 1 Department of Internal Medicine and Cardiology, “Prof. Dr. Th. Burghele” Clinical Hospital, 061344 Bucharest, Romania; ondin.zaharia@umfcd.ro (O.Z.); tiberiu.nanea@umfcd.ro (I.T.N.); 2 Department of Cardio-Thoracic Pathology, Cardio-Thoracic Pathology, Faculty of Medicine, Carol Davila University of Medicine and Pharmacy, 050471 Bucharest, Romania; 3 Department of Cardiology I, Central Military Emergency Hospital “Dr Carol Davila”, 030167 Bucharest, Romaniasilviu.dumitrescu@scumc.ro (S.I.D.); 4 Department of Morphological Sciences, Cell and Molecular Biology and Histology, Carol Davila University of Medicine and Pharmacy, 050474 Bucharest, Romania; sanda.cretoiu@umfcd.ro; 5 Department of Cardiology, Faculty of Medicine, Titu Maiorescu University, 040441 Bucharest, Romania

**Keywords:** heart failure, inflammation, proinflammatory cytokines, TNF-α, CRP, fibrinogen, IL-1, IL-6, iNOS, myeloperoxidase

## Abstract

Chronic heart failure is a terminal point of a vast majority of cardiac or extracardiac causes affecting around 1–2% of the global population and more than 10% of the people above the age of 65. Inflammation is persistently associated with chronic diseases, contributing in many cases to the progression of disease. Even in a low inflammatory state, past studies raised the question of whether inflammation is a constant condition, or if it is, rather, triggered in different amounts, according to the phenotype of heart failure. By evaluating the results of clinical studies which focused on proinflammatory cytokines, this review aims to identify the ones that are independent risk factors for heart failure decompensation or cardiovascular death. This review assessed the current evidence concerning the inflammatory activation cascade, but also future possible targets for inflammatory response modulation, which can further impact the course of heart failure.

## 1. Introduction

Heart failure (HF) is a diagnosis which appears in the context of a systolic or/and diastolic dysfunction of the left ventricle (LV) [1]. Characteristic signs and symptoms of the disease can be considered: shortness of breath with orthopnea, lower limb oedema and reduced exercise capacity, accompanied by jugular vein distension and ventricular gallop, with an added S3 (third sound) or S4 (fourth sound) [2]. After physical examination, the diagnosis is confirmed by several investigations: biological (NT-proBNP) and imagistic (echocardiography, cardiac-MRI) [3].

Although many new active substances and invasive procedures have been developed in the last decades with great results shown by clinical randomized trials (CRT), the prognosis of the patients diagnosed with chronic heart failure remains poor. The rate of death during the first admission for heart failure is between 2% and 17%. The mortality rate within 1 year from the admission is between 17% and 45%, and above 50% during the first 5 years since the diagnosis [4].

Chronic heart failure (CHF) brings an excessive burden on societies and healthcare systems worldwide, not only by the mortality it generates, but also on an even greater scale, by the morbidity it brings, caused by prolonged hospitalization and high rates of readmission [5].

In the last century, the causes and mechanisms considered to generate HF were the theory of inotropic dysfunction and volemic overload [6]. The present paradigm is around the neurohormonal system activation, which appears in response to the severely altered working conditions of the heart (increased intracavitary pressures and perfusion mismatch) [6]. The pharmacological treatment endorsed by current guidelines consists of substances, which aim to inhibit the neurohormonal paths of activation, an effect which can also produce reverse-remodelling of the geometry of heart chambers [7].

The CRTs showed great benefit on the endpoints of mortality and risk of readmission for CHF, with statistically significant results especially in the patients with reduced ejection fraction (HFrEF—heart failure with reduced ejection fraction) [8]. These classes of medical therapy are the angiotensin-converting enzyme inhibitors (ACEI), beta blockers (BB), mineralocorticoid receptor antagonists (MRA), inhibitor of angiotensin II receptor and neprilysin (ARNI) [9]. Another new class of drugs joined the well-established classes, according to the last guideline for the treatment of CHF of the European Society of Cardiology (ESC) 2021: the SGLT2i (sodium-glucose cotransporter 2 inhibitors) [3]. This hypoglycaemic oral antidiabetic type of drug showed a decrease in the mortality and/or readmission risk composite endpoint both in the HFrEF and HFpEF (heart failure with preserved ejection fraction), a first-of-a-kind result [10,11]. This aspect is of great interest because more than 50% of the patients admitted with CHF prove to have a HFpEF phenotype [12,13].

**Figure 1:**
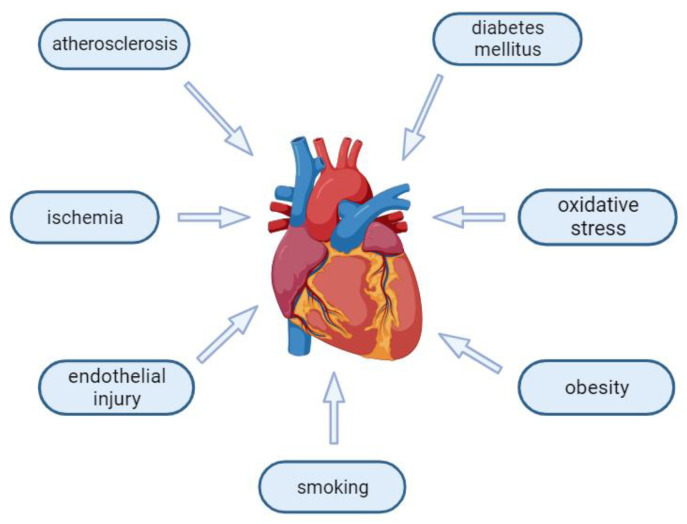
Heart failure risk factors. Created using BioRender.com (accessed on 5 November 2023).

Systemic inflammation is recognized to be correlated with CHF, being considered to have a role in the development, progression and furthermore, complication of this disease [14]. At the same time, the inflammatory markers are considered a prognostic factor of poor outcomes, independent of the usual HF indicators of decline: LVEF (left ventricle ejection fraction) or NYHA (New York Heart Association) functional class [15]. The levels of non-specific inflammatory markers and proinflammatory specific cytokines in the context of CHF are significantly lower than the ones appearing in the course of infections or autoimmune diseases [16]. This raises the suspicion that a low-grade state of chronic inflammation is the constant hit taken by the cardiomyocytes, contributing to the maintenance and evolvement of CHF, with repeated episodes of acute decompensation [17]. Risk factors associated with the development of heart failure can be seen in Figure 1.

The level of inflammatory cytokines and immune system cells found to be activated in CHF patients reached 57% of the enrolled patients in the RELAX (Phosphodiesterase-5 Inhibition to Improve Clinical Status and Exercise Capacity in Diastolic Heart Failure with Preserved Ejection Fraction) trial. These enrolled patients had elevated C-reactive protein (CRP) levels [18]. The median of high-sensitivity CRP (hsCRP) was 6.6 mg/L in the patients with stable chronic HF with reduced and preserved ejection fraction (EF) and 8.5 mg/L in the TIME-CHF (Trial of Intensified versus Standard Medical Therapy in Elderly Patients with Congestive Heart Failure) trial [14]. The level of hsCRP was even higher in patients with acute HF, as shown in the ASCEND-HF (Acute Study of Clinical Effectiveness of Nesiritide in Decompensated Heart Failure) trial [19].

Inflammation is a major factor in the development and progression of CHF in every phenotype of CHF: HFrEF, HFmrEF (heart failure with mildly reduced ejection fraction) and HFpEF [20]. However, two recent biomarker profile analyses demonstrated that there is a greater link between inflammatory markers and HFpEF, as shown in the COACH (Counseling in Heart Failure) and BIOSTAT-CHF (Biology Study to Tailored Treatment in Chronic Heart Failure) trials [21]. This can be explained by the fact that HFpEF patients usually have a longer list of concomitant chronic diseases, such as diabetes mellitus, chronic obstructive pulmonary disease, obesity and chronic kidney disease, which contributes to greater levels of inflammation. The inflammation is produced mainly by activation of endothelial microvascular cells of the immune system, increased reactive oxygen species (ROS) and a reduction in nitric oxide (NO), which is produced mainly at the site of endothelial cells by the eNOS (endothelial nitric oxide synthases) [22]. This chronic inflammation has a chemotaxis effect on monocytes, which gather in high numbers in the myocardium, in order to then be transformed into proinflammatory macrophages (M1) [23].

HFrEF had lower average inflammatory marker levels, but greater heart failure specific elevated markers [24]. However, although the level of inflammatory activation is lower than in the HFpEF phenotype, the injury of cardiomyocytes triggers the proinflammatory cytokines and chemokines, which is followed by neutrophil and monocyte infiltration in the myocardium [25]. The next phase in the inflammatory process, called the reparative phase, involves phagocytosis of apoptotic and necrotic cells, with T and B lymphocyte chemotaxis, collagen synthesis from myofibroblasts, leading to LV remodelling and the release of anti-inflammatory and pro-resolving molecules (TGF-β, lipoxins and IL-10). The last phase, maturation, is marked by the death of the reparative cells and scar maturation [25]. The chronic inflammation stimulus can be from repeating myocardial injury, with consequent inflammatory cascade activation, or because of the neurohormonal systems activation (sympathetic nervous system, renin-angiotensin-aldosterone system) [26]. Another cause of chronic inflammation is the disturbance of inflammatory resolution. Two groups of researchers found that patients with CHF with III or IV NYHA functional class had decreased plasma and urinary lipoxins levels compared to CHF with I or II NYHA functional class patients. At the same time, patients with CHF had lower levels of resolvin D1, as opposed to healthy age-matched individuals [27,28].

Chronic, low-grade inflammation, induces significant effects on myocardial structure and function. The inflammatory state is a frequent feature in cases of HFpEF. The impact of inflammation on HFpEF evolution can be attributed also to the left atrial stiffening which amplifies with age, irrespective of the associated LV hypertrophy and concentricity. As left atrial stiffening can be responsible for the aggravation of congestion, it can lead to a decline in the functional status of HFpEF patients [29]. Reduction of disease severity and evolution in HFpEF patients was noted after the suppression of certain inflammation pathways.

In HFpEF, high seric levels of inflammatory biomarkers can be found, such as IL-1β, IL-6, IL-10, CRP, TNF-α and myeloperoxidase [30]. Inflammasome signalling is involved in the interplay between chronic inflammation and CVD (cardiovascular disease) development. Moreover, evidence suggests that systemic inflammation can be triggered by glucose-mediated redox stress insulin resistance and by the activation of the vascular inflammasome in type 2 diabetes mellitus patients [30].

Low grade inflammation, evaluated by hsCRP, was an independent risk factor for vascular and all-cause mortality in type 2 diabetic patients in the study conducted by Sharif et al. This draws attention to a potential treatment target to diminish the cardiovascular risk in diabetic patients [31].

High arterial pressure leads to ROS production and further generation of proinflammatory cytokines in renal or pulmonary vessels in HFpEF patients [32] or animal subjects [33].

In obese patients, macrophage and monocyte infiltration in visceral adipose tissue augments cytokine release, which can further amplify the inflammatory state [30]. 

**Figure 2:**
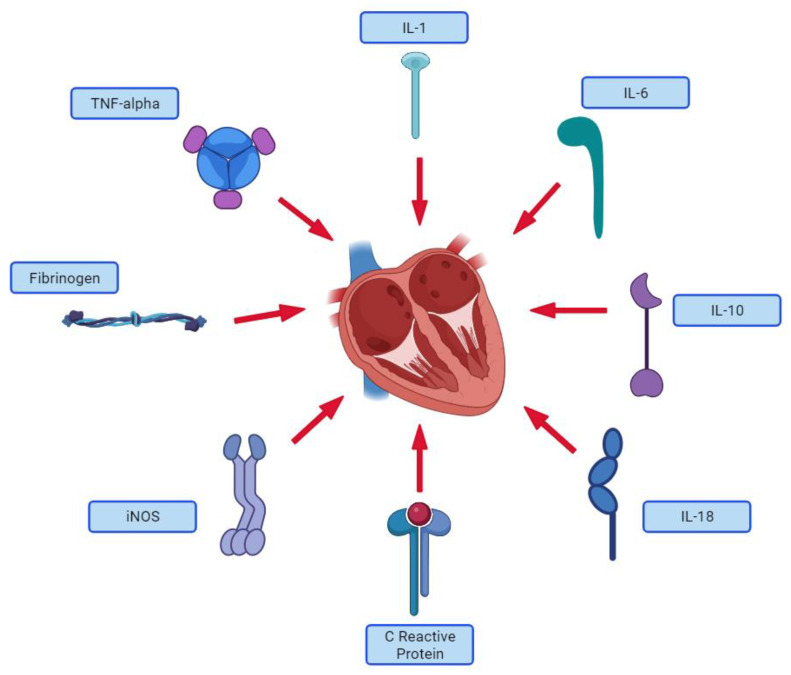
Proinflammatory cytokines associated with heart failure. Created using BioRender.com (accessed on 5 November 2023).

In Figure 2, the major proinflammatory cytokines contributing to heart failure are presented.

The aim of this review is to present the inflammatory activation cascade which further leads to cardiac injury, inducing and aggravating chronic heart failure and to discuss clinical trials evaluating specific cytokines, but also to underline future therapeutic options for inflammation in HF.

## 2. Inflammatory Mediators Taking Part in the Development and Evolvement of Heart Failure

### 2.1. TNF-α

TNF-α, a pivotal proinflammatory cytokine, is the most studied one in HF, since it can be produced by many types of cells: cardiomyocytes, macrophages, vascular cells and mast cells [34]. After binding to the cell membrane specific receptors (TNFR1 and TNFR2), TNF-α has a negative inotropic effect on cardiomyocytes, by reducing the cytosolic level of Ca^2+^ [35]. At the same time, TNF-α induces synthesis of other proinflammatory cytokines (inducible NO synthase, reactive oxygen species with mitochondrial DNA damage), apoptosis and extracellular matrix alteration and promotes interaction between endothelial cells and leukocytes at the site of microcirculation [34]. Also, high levels of TNF-α enhance protein synthesis and hypertrophy of cardiomyocytes by the production of reactive species of oxygen, which further decrease the contractility of the heart [36].

The sympathetic nervous system, one of the neurohormonal pathways which leads to cardiac remodelling, has its function impaired by TNF-α, resulting in β receptor dysfunction [37]. This triggers, in response, an excessive release of catecholamines, which produces a positive feedback increase in the levels of seric TNF-α, creating a perpetual cycle [38].

The proinflammatory TNF-α has significant interactions with the sympathetic system, its interaction with β receptors leading to a negative inotropic effect, both in vitro and in vivo [39,40].

In heart failure patients, the sympathetic overdrive and high levels of proinflammatory cytokines are in a vicious circle, representing, however, a hallmark of this disease [41,42].

Although this cytokine has been proven to take part in the inflammatory infiltrate of both HFrEF and HFpEF patients [43], efforts from clinical trials to inhibit it have been without evident success [17,44]. This can be explained by the fact that in other experimental studies, in ischemia-reperfusion injury scenarios, a small quantity of TNF-α had a protective cardiac effect, while only high levels of TNF-α had negative effect on cardiac function and remodelling [45,46].

The trials which tried to see the effect of the soluble receptor of TNF-α (etanercept) in chronic heart failure patients (RENEWAL and RECOVER), were stopped early in the process because of the negative results, in comparison to the placebo arm [17]. The same worsened prognosis was also observed in the case of the TNF-α monoclonal antibodies (infliximab) trial ATTACH [44]. There is a well-established direct relationship between the circulating levels of TNF-α and mortality in the patients which suffer from heart failure [41,47]. Since TNF-α is associated with a serious decrease in survival, it can be considered for risk assessment in HF patients with both HFpEF and HFrEF [47]. 

### 2.2. IL-1

IL-1 is one of the most important cytokines in the initiation of inflammation in HF [48]. The elevated levels of IL-1 are seen in patients with chronic HF, irrespective of etiology (ischemia, arterial hypertension, valvular disease, cardiomyopathy, arrhythmia) [49]. It represents a family with 11 members including IL-1α, IL-1β, IL-18 and IL-33 [50]. If IL-1α or IL-1β bind to the IL-1R1 (IL-1 type 1 receptor), this initiates the inflammatory process, while binding to IL-1R2 (IL-1 type 2 receptor) stops the triggering of the inflammatory response [51]. The synthesis of the active form of IL-1β is directly dependent on the caspase-1 enzyme, which is also at the expense of NLRP3 inflammasome (an intracellular sensor activated in the onset of danger-associated signals) [52,53]. NLRP3 gets activated in the cardiac fibroblasts and cardiomyocytes when there is a myocardial injury and this can also explain the proinflammatory response that appears post-myocardial infarction that further inflicts cardiac injury [54]. IL-1 also generates cardiac impairment and remodelling by reducing the capacity of the LTCC (Ca^2+^ channels type L) to respond to the sympathetic nervous system (β1 adrenergic) stimulus [48]. This cytokine, which can be produced by cardiomyocytes, immune cells, endothelial cells and fibroblasts, also reduces the expression of genes that promote calcium homeostasis and induces cardiomyocyte apoptosis, activation of endothelial cells and leukocytes, leading to cardiac fibrosis, micro arterial stiffness and inflammation [50]. One of the cytokines related to IL-1 is CRP, which is an independent predictor for cardiac decompensation in acute or chronic heart failure [55]. IL-1β can also disrupt mitochondrial energy production, which translates as dysfunctional myocardial inotropism [48,56].

This energetic “outage” produced by IL-1 is generated in part through an increase in nitric oxide synthase activity in the cardiac cells with a consequence of reduced myocardial contraction [57]. In the animal model trials, the mice that were injected with plasma rich in IL-1, from acute decompensated heart failure patients, had systolic and diastolic dysfunction with a decrease in contractile reserve [58]. However, the mice that were previously treated with the IL-1 human antagonist (anakinra) prior to plasma injection, were protected against cardiac impairment, leading to the conclusion that IL-1, and more specifically IL-1β, acts as a cardiodepressant agent [59]. The same antagonist of IL-1 was studied in the D-HART trial in a total of 12 chronic heart failure patients which were assigned to anakinra or placebo [48]. The primary endpoint of peak oxygen consumption was measured initially, at 14 and 28 days, showing a significant increase in peak oxygen consumption in patients receiving anakinra correlated with a statistically significant decrease in C-reactive protein seric levels [60].

It is known that IL-1 is a mediator in HF by diminishing cardiac contractility and promoting cardiomyocyte hypertrophy and apoptosis. However, there is also evidence that IL-1 is involved in atherothrombosis, by stimulating the development of atheromatous lesions, it promotes vascular inflammation and favours plaque vulnerability. In acute scenarios, after myocardial infarction, IL-1 is involved in the inflammatory response and adverse cardiac remodelling through amplifying matrix metalloproteinase expression [61]. 

### 2.3. IL-6

IL-6 is an important inflammation mediator, which can be regarded as a possible future biomarker for the development of HFpEF. In HF, oxidative stress is a strong inducer for the production of IL-6 [62]. Ischemia and hypoxia lead to IL-6 auto or paracrine binding to its receptor (receptor-coupled protein gp130), then following the JAK/STAT3 signalling pathway, leading to abnormal endothelium-dependent vasodilatation and muscular atrophy [63].

In HF, various cells can produce inflammatory mediators, such as IL-6, which can have several effects: systolic dysfunction, diastolic dysfunction, ventricular dilatation, cardiomyocyte hypertrophy, apoptosis [49] and lower coronary flow reserve [63]. IL-6 influences the inflammatory process, favouring ventricular remodelling, which is responsible for the debut, but also for the aggravation, of HF symptoms [64].

IL-6 has a multitude of effects on cardiac cells, some of which can lead to a myocardial phenotype very similar to that of the hypertensive heart (hypertrophy, fibrosis, diastolic dysfunction, further favouring HFpEF) [65,66].

The interest in anti-IL-6 drug development is increasing, since higher levels of IL-6 were statistically significantly associated with a higher risk of HFpEF development [67]. On the other hand, de Boer and colleagues indicated that IL-6 was associated with new-onset HF, IL-6 being evocative for HFrEF [68]. However, these differences between correlation with HFrEF and HFpEF may come from the dissimilarity between cohorts. 

In the BIOSTAT-CHF cohort, HFpEF was an independent predictor of high IL-6 concentrations. In more than half of the patients involved, high IL-6 concentrations were associated with lower EF, iron deficiency and atrial fibrillation [69].

In patients with decompensated HFpEF, IL-6 was a predictor of all-cause mortality, cardiovascular mortality and HF hospitalization, according to Mooney et al. [70].

By downregulating SERCA2 gene expression, IL-6 and TNF-α can cause diastolic dysfunction by diminishing diastolic calcium reuptake, further leading to myocardial contractility impairment [71].

There is evidence that high concentrations of IL-6 can be found in patients with LV dysfunction, even when there is no clinical syndrome of HF. IL-6 may play a role in the evolution from asymptomatic LV dysfunction to symptomatic LV dysfunction, representing a promising biomarker for patients at risk of developing clinical HF, especially HFpEF [67].

IL-6 exerts negative effects on renal function by acting on the distal tubule (epithelial sodium channels), altering the process of natriuresis [72]. It is important to underline that another renal repercussion of IL-6 action can be diuretic resistance [73]. 

Hemodynamic worsening of the evolution of advanced HF patients was associated, according to Gabriele et al., with high levels of IL-6 and IL-6R mRNA [74].

### 2.4. IL-8

IL-8 (or CXCL8) can be produced mostly by macrophages and monocytes, but also by neutrophils, epithelial cells, fibroblasts, smooth muscle cells and endothelial cells, when triggers such as ischemia, hypoxia or shear stress are present [75]. 

IL-8 recruits, through chemotactic effect, monocytes and neutrophils, the main constituents of the acute inflammatory response. Besides recruitment, IL-8 also favours the activation of monocytes and neutrophils [76].

A special characteristic of IL-8 is its longevity, since it is produced in the first steps of the inflammatory response, but stays active for days, even weeks. It is resistant to temperature and proteolytic enzymes and relatively resistant to acidic environments, making it very useful in places of acute inflammation. IL-8 is very sensitive to oxidants, therefore antioxidants can significantly lower IL-8 gene expression [77].

IL-8 is involved in atherosclerosis. It can be found in high amounts in atherosclerotic lesion macrophages, in vascular injury sites and in fibrous plaques [76]. 

It was reported that angiotensin II increased IL-8 production, while fluvastatin diminished both basal and angiotensin II-induced IL-8 production in human vascular smooth muscle cells [78]. 

IL-8 is also involved in the pathogenesis of hypertension, being expressed in high concentrations in aortic tissue and vascular smooth muscle cells of hypertensive animal model [79].

Simonini et al. indicated that IL-8 is a significant mediator of angiogenesis in human coronary atherosclerosis, which may contribute to atherosclerotic plaque formation through its angiogenic properties [80].

A report of the potential role of IL-8 as a biomarker for chronic HF was highlighted in the results from the CORONA study, which evaluated patients aged over 60 years, with chronic HF of ischemic cause, with II-IV NYHA functional class and LVEF under 40%. The primary outcomes were a composite of CV mortality, non-fatal myocardial infarction and non-fatal stroke, and secondary outcomes were any coronary event, sudden cardiac death, ventricular defibrillation by implantable cardioverter-defibrillator, resuscitation after cardiac arrest, hospitalization for unstable angina pectoris, all-cause mortality, CV mortality and a composite endpoint of worsening HF hospitalization or CV mortality [81].

There was a statistical association between IL-8 and outcomes. However, IL-8 added information independent of hsCRP, which further underlines that they may represent different inflammatory pathways in chronic HF. NTproBNP and IL-8 were significantly associated with both cardiac and non-cardiac deaths. IL-8 was a consistently independent and significant predictor of outcomes after statistical adjustment for NTproBNP [81].

IL-8 is found in high concentrations in CHF and is associated with adverse outcomes [81,82], and it is a predictor of the development of HF in patients with myocardial infarction and percutaneous intervention [83].

### 2.5. IL-10

IL-10 is a major anti-inflammatory cytokine. Inflammation has essential roles in the development of cardiac hypertrophy and evolution to HF. IL-10 can be expressed in the cardiac tissue and may have an essential role in cardiac remodelling. For this reason, signalling modulated by IL-10 could become a promising target for controlling pathological cardiac hypertrophy [84].

Supporting the impact of IL-10 on cardiac remodelling is the work of Jung M. et al., which concludes that in vivo infusion of IL-10 after MI can improve the LV microenvironment, decrease inflammation and favour cardiac wound healing by stimulating M2 macrophage polarization and fibroblast activation [85].

Verma and colleagues showed that IL-10 treatment could be a potential therapeutic target in limiting the evolution of cardiac remodelling induced by pressure overload [86].

IL-10 can suppress inflammation, improve LV function and attenuate LV remodelling after MI by reducing fibrosis through inhibition of HuR (cytokine mRNA stabilizing protein) and activation of signal transducer and activator of transcription 3 (STAT-3), by increasing capillary density [87].

The antiatherosclerotic effect of IL-10 was intensely discussed. IL-10 can have effects on macrophages and T cells, modulating several cellular processes, which may interfere with the formation, evolution and stability of the atherosclerotic plaque. IL-10 was associated with low signs of inflammation, but was also a protective factor against environmental pathogens which can promote atherosclerosis in animal subjects [88]. 

In the case of ischemia-reperfusion injury, TNF-α is increased, which further initiates and sustains inflammation as well as cardiac injury. IL-10, being an anti-inflammatory cytokine, inhibits signalling pathways which participate in the pathogenesis of HF controlled by TNF-α [89]. 

Diminished seric concentrations of IL-10 were identified in patients with advanced CHF [90]. 

In special populations, such as patients suffering from chronic kidney disease (CKD), IL-10 was observed to increase along with the reduction of kidney function. Elevated IL-10 concentrations were associated with the risk of CV events [91].

Barcelos and colleagues evaluated the association between IL-10 and coronary artery disease in patients suffering from metabolic syndrome. In this category of patients, high IL-10 concentrations were associated with a lower incidence of severe coronary artery disease. This suggests a protective effect given by the anti-inflammatory activity even when there are significantly high concentrations of proinflammatory cytokines [92].

IL-10 proved to be cardioprotective in diabetic MI through the upregulation of heme clearance pathways. IL-10 lowered the myocardial infarct size and improved cardiac function in diabetic animal subjects, improved capillary density and lowered apoptosis rate and inflammation in the marginal zone of the infarct [93]. 

### 2.6. IL-18

IL-18, also named interferon gamma (IFN-γ) inducing factor, is a proinflammatory cytokine, belonging to the IL-1 cytokine superfamily. It has effects on immunity and the infectious and inflammatory response of the host, due to the production of IFN-γ. However, it also possesses other effects, independent of IFN-γ. IL-18 can be produced, as a response to injury, by infiltrated neutrophils, macrophages, endothelial cells, smooth muscle cells and cardiomyocytes. It is produced in an inactive form (pro-IL-18), being converted into the active form by caspase 1 (IL-1beta converting enzyme) [94].

High concentrations of IL-18 have been detected in myocardial tissue and circulation after MI and in sepsis [94]. 

IL-18, being a proinflammatory cytokine, is also involved in atherosclerosis. Jia et al. concluded that both IL-6 and IL-18 were associated with global CV disease and death [95].

Plausible molecular mechanisms regarding IL-18-induced myocardial injury can be represented by the promotion of inflammation, enhanced apoptosis, hypertrophic effect on cardiomyocytes, effects on mitogen activated protein kinase activation and alterations of the intracellular calcium transport and concentrations [94].

In patients with congestive HF, a high secretion of IL-18 is induced and is correlated with the severity of myocardial damage and dysfunction, according to Seta et al. [96].

O’Brien and colleagues evaluated IL-18 as a potential therapeutic target in acute MI and HF. In animal subjects, it is known that IL-18 influences cardiomyocyte hypertrophy and favours contractile dysfunction and extracellular matrix remodelling in cases of acute MI or pressure overload. In human subjects, elevated IL-18 levels were correlated with a higher risk of appearance and progression of HF and with a worse prognosis in patients with already established CVD [97]. 

Inhibition of IL-18 alters not only the pathological, but also the physiological hypertrophy response in cases of high pressure, which can result in improper remodelling [96].

Genetic deletion or neutralization in animal subjects of IL-18 lowered the rate of myocardial hypertrophy in cases of pressure overload [97,98].

IL-18 displays effects on both systolic and diastolic functions of the heart. The rapid negative inotropic effect of IL-18 implies that blocking IL-18 may represent an important treatment for acute decompensated HF or chronic, symptomatic HF [97,99].

Inflammatory cytokines are involved in the progression of HFpEF, considering that in this phenotype significant fibrosis and hypertrophy can be found [60]. IL-18 could be a possible treatment target for HFpEF since it has pro-hypertrophic and profibrotic effects [97,100,101]. 

There is evidence suggesting that plasma IL-18 concentrations are associated with coronary events [102,103].

### 2.7. Fibrinogen

Fibrinogen, a major acute phase protein, is widely recognized as a strong contributor to cardiovascular risk, high concentrations being associated with coronary heart disease, incident stroke, development of peripheral artery disease and total mortality. In inflammation, the abundance of cytokines elevates plasmatic concentrations of fibrinogen. Fibrinogen has several roles, such as influencing endothelial function, favouring smooth muscle cell proliferation and migration, modulating the interaction between plasmin and the corresponding receptor, creating the substrate for thrombin, constituting the final step in the coagulation process and being involved in platelet aggregation [104,105].

In over 2000 subjects from the Framingham Offspring Population (cycle 5), fibrinogen was associated with traditional cardiovascular risk factors, levels of fibrinogen being higher among individuals with known cardiovascular disease, compared to those without cardiac afflictions [106].

Even in healthy individuals, fibrinogen is a considerable risk factor for cardiovascular disease. In patients who suffered a coronary event, high levels of fibrinogen are a risk factor for recurrence of myocardial ischemia or a risk factor for mortality, predicting fast-developing atherosclerosis. Acute or chronic elevations of fibrinogen concentrations can favour atherosclerotic events by fibrinogen infiltrating the wall vessels, rheological effects caused by high blood viscosity, augmented thrombocyte aggregation, thrombus development and accentuated fibrin formation [107]. 

There is evidence that plasmatic fibrinogen concentrations are positively associated with cardiovascular events and are a contributor to atherosclerotic events [107]. 

The role of fibrinogen in the prognosis of critically ill patients with acute decompensation of chronic HF was evaluated by Meng et al. Their work concluded that high fibrinogen levels (over 284 mg/dL) predicted, independently, the mortality in the previously mentioned category of patients [108]. 

### 2.8. CRP

Continuous, but low-grade inflammation is present in the context of HF. One can aim to discuss the role of CRP, as an inflammatory marker, in the pathogenesis and development of HF.

CRP has several roles in the mechanism of atherogenesis, such as increasing LDL uptake and oxidation, inhibition of NO production, upregulation of the expression of adhesion molecules, inhibition of fibrinolysis (by amplifying the expression of PAI-1), inducing complement activation and favouring monocyte infiltration into the vascular wall [109]. 

In patients with acute coronary syndromes, high CRP levels at admission are associated with poorer short- and long-term prognosis. CRP value on admission shows the baseline inflammatory status of the subject, elevated CRP concentrations in patients with acute coronary syndromes being linked to more cardiovascular complications during follow-up [109]. The more elevated the CRP levels, the greater the chances for severe acute coronary syndrome, ventricular remodelling, lower EF, cardiac rupture, HF and cardiac death. It is interesting to note that in STEMI patients, peak CRP levels were higher when compared to NSTEMI patients, drawing attention to the possible role of CRP in risk stratification after myocardial infarction [110].

Microvascular inflammation is involved in the pathogenesis of HFpEF. In these patients, higher levels of CRP were associated with higher comorbidity burden and markers of disease severity, although normal CRP levels were observed in 40% of subjects [111].

In HFpEF, elevated CRP concentrations were associated with considerable comorbidity burden [111].

A systematic review and a meta-analysis conducted by Lakhani et al, which assessed the diagnostic and prognostic role of CRP in HFpEF, underlined that CRP could be used as a biomarker to predict the development of HFpEF and the long-term clinical outcomes in this category of patients [112]. 

Inflammatory markers, such as IL-6, TNF-α and CRP were independently associated with incident HF. IL-6 and CRP were associated with HFpEF, but not with HF with reduced or moderately reduced EF. This conclusion can underline that the activation of IL-6 and the CRP pathway could be specifically attributed to HFpEF [113].

When assessing the predictive value of CRP in patients with HFpEF, it proved to be an independent and strong predictor of mortality in patients suffering from HFpEF. CRP added prognostic value to NTproBNP, the highest mortality risk being in the group with both the highest CRP and highest NTproBNP concentrations. These results draw attention to the immunology phenomena which negatively impact the evolution of HFpEF [114].

Regarding the distribution depending on gender, CRP was strongly and independently associated with HF in men. However, for women, the association of CRP and HF was weaker and disappeared after accounting for CV risk factors [115]. 

In patients already diagnosed with CV disease, CRP proved to be an independent risk marker of incident HF (as defined by the first hospitalization for HF), according to Burger et al. [116].

A systematic review conducted by Araujo and colleagues evaluated the link between hsCRP (as a marker of low-grade inflammation) and the prediction of HF in general and high-risk populations. They also evaluated the prognostic role of hsCRP in patients already diagnosed with HF. Past studies indicated that hsCRP is a powerful independent predictor of acute myocardial infarction and CV death. HsCRP was associated with incident HF in high-risk but also general populations and it possessed prognostic information in patients suffering from HF. Although different cut-off values for hsCRP were assessed in the studies included in the analysis, hsCRP had an important prognostic power in cases of both incident HF and already diagnosed HF in various populations [117]. 

Plasmatic levels of hsCRP were associated with more congestion and a worse prognostic in patients with chronic HF [118]. In ambulatory patients suffering from HF, higher hsCRP plasmatic concentrations were a strong mortality predictor and pointed out patients with higher natriuretic peptides, who were more prone to die of non-CV causes [118].

CRP concentrations are associated with prognosis in CV diseases, acute coronary syndromes included. CRP levels at admission are associated with hospital mortality in acute decompensated HF patients and with a high risk of long-term mortality [119].

A prospective study on STEMI patients who underwent coronary angioplasty indicated that peak CRP levels in these patients predicted the emergence of HF. Peak CRP levels (usually reached after 48 h) were also a strong predictor of global and CV mortality in the first year after the acute coronary event. A positive correlation between maximum CK levels and peak CRP and a negative correlation between LVEF and peak CRP were observed [120].

When assessing a possible connection between central sleep apnoea and CRP levels in patients with chronic HF, it was observed that severe central sleep apnoea in HF patients is associated with high seric concentrations of CRP, suggesting a negative prognostic marker for chronic HF in this category of patients [121].

The prognostic values of CRP and statins in patients with HFrEF and HFpEF were evaluated in a study conducted by Park et al. The results indicated that CRP was a very good prognostic marker for HFrEF, HFmrEF and HFpEF, and also that statins could be beneficial in cases of HF with high CRP concentrations [122].

HsCRP has been associated with outcomes in adult congenital heart disease (ACHD). Geenen and colleagues evaluated approximately 600 patients with ACHD over a mean period of 5.9 years. Higher baseline hsCRP was independently associated with greater risk of death or HF. Therefore, according to their results, it can be stated that hsCRP has incremental prognostic value for the risk of death or HF, independent of NTproBNP and hs troponin T. The clinical decline of patients with ACHD was anticipated by elevated hsCRP concentrations, which were increased before symptoms of HF or death [123].

### 2.9. iNOS (Inducible Nitric Oxide Synthase)

Nitric oxide (NO) is a diffusible free radical gas with a very short half-life. It is synthesized from l-arginine through the catalytic reaction of nitric oxide synthases (the neuronal type 1 isoform—nNOS or NOS_1_; the inducible type 2 isoform—iNOS or NOS_2_; and the endothelial, type 3 isoform—eNOS or NOS_3_). The activity of nNOS and eNOS is triggered, therefore it is transient. On the other hand, iNOS activity is sustained, as it does not depend on stimulating agonists and calcium [124].

nNOS is found in nerve endings (neurotransmission of norepinephrine) and eNOS in endothelial cells, endocardial cells and cardiomyocytes [124].

The effects of NO in the human heart include the inhibition of the positive inotropic effect as a result of beta-adrenergic stimulation in cases of LV dysfunction or severe HF [125,126]. 

The inducible nitric oxide synthase (iNOS or NOS_2_) is normally expressed in low concentrations in myocardial tissue. In specific cases, such as inflammation or ischemia, significant amounts of NO are generated, after the activation of iNOS [127].

NO has roles in preserving vascular tone and preventing platelet aggregation or adhesion. Therefore, altered NO production will lead to inflammation and cellular destruction [128]. 

iNOS activation leads to large quantities of NO, which can be cytotoxic or inhibit myocardial contractility [129].

Evidence suggests that iNOS is also associated with significant oxidative stress and insulin resistance [130], both of which are known to be involved in the pathophysiology of HFpEF. Animal studies indicate that stress produced by iNOS leads to HFpEF phenotype progression, while its inhibition leads to an improvement of HFpEF in mouse models [131]. Considering the evidence from animal studies, it is promising to evaluate a possible therapeutic role of iNOS inhibition in HFpEF [128].

Cardiac iNOS activity in conditions involving myocardial and systemic inflammation, such as in severe HF patients and septic shock, can reduce the response to beta-adrenergic activation. NO has a negative effect on inotropism after beta-adrenergic stimulation through cGMP inhibition of calcium influx caused by voltage dependent Ca^2+^ channels (L-type) [132]. 

NO can lower beta-adrenergic stimulation in HF cases, while elevated cardiac iNOS activity is linked to the early debut of relaxation [124]. 

Chronic HF is associated with iNOS expression and activity. In cases of dilated cardiomyopathy, iNOS is expressed in cardiomyocytes, along with TNF-α [133].

Significant expression of iNOS can be noted in patients with ischemic cardiomyopathy, suggesting that iNOS myocardial expression is a consequence of HF and it is not necessarily related to the cause of HF [124,134]. 

There is, however, conflicting evidence regarding eNOS. Its activity was noted to be high in some cases of HF, but other studies identified it as diminished [124].

Zhang et al. underlined that iNOS expression has a role in the maladaptive response (myocardial hypertrophy and cardiac chamber dilation) caused by pressure overload. They also indicated that iNOS inhibition could be a solution for systolic-induced cardiac dysfunction [135].

Transgenic models imply that intense iNOS activity can lead to significant structural and functional cardiac changes. Mungrue and colleagues indicated that there is a correlation between chronic overexpression of iNOS and cardiac dilatation, conduction abnormalities, sudden cardiac death and HF. Accentuated cardiac iNOS activity has pathogenic potential, implying that selective inhibition of iNOS may be an important therapeutic strategy for cardiovascular afflictions [136].

Liu et al. concluded that iNOS did not appear to be a pathological mediator of HF, but the absence of iNOS improved cardiac reserve after ischemic events (such as myocardial infarction), especially when constitutive NOS isoforms were blocked. Further studies are needed to establish if diminished oxidative stress or other adaptive mechanisms could be responsible for this effect [137].

Ferreiro and colleagues evaluated the expression of iNOS in HF caused by ischemic disease. They concluded that HF downregulated eNOS activity and expression in the myocardial tissue of patients with HF and an EF below 35%, but they also emphasized that iNOS activity and expression were higher in HF cases caused by ischemic disease [138].

In patients with advanced, refractory HF, iNOS protein expression was elevated before heart transplantation. Mechanical unloading with ventricular assist devices lowered iNOS protein concentrations, in concordance with a lower rate of cardiomyocyte apoptosis [139].

### 2.10. Myeloperoxidase (MPO)

MPO is a leukocyte-derived enzyme, a heme peroxidase, mainly expressed by neutrophils, which belongs to the innate immune response. MPO-derived oxidants are responsible for tissue destruction in inflammatory scenarios [140,141].

MPO has the ability to generate reactive species, with important roles in the innate host immunity and therefore antimicrobial activity. High circulatory levels of MPO are associated with inflammation, increased oxidative stress, poor prognosis and high risk of CVD-related mortality [140].

MPO can be regarded as an important target for cardiovascular protection. Circulating MPO can be regarded as an indicator of high risk in patients with acute coronary syndromes, atherosclerosis, heart failure, hypertension or stroke [140].

The role of MPO in atherosclerosis can be suggested by the MPO catalysed reactions, with pro-atherogenic effects, transforming MPO and its inflammatory pathways into potential therapeutic targets for the prophylaxis of atherosclerosis [141].

In chronic HF patients, MPO plasmatic levels were a predictor of adverse clinical outcomes, being also associated with the severity of HF, according to Tang et al. [142].

The risk prediction for adverse clinical events in chronic HF had a stepwise increase when patients were stratified according to hsCRP, MPO and NTproBNP. Moreover, hsCRP and MPO delivered complementary prognostic value for chronic systolic HF patients involved in the study of Wilson Tang and colleagues [143].

Another study which evaluated chronic HF patients underlined the differences in MPO concentrations between HF patients and healthy subjects. A positive correlation was identified between MPO and chronic HF severity, as MPO was significantly higher in patients who had higher mortality rates [144].

Elevated systemic MPO levels proved to be independently associated with the risk of HF development over a long follow-up for more than 3000 healthy elderly subjects, especially in subjects without traditional CV risk factors [140,145].

In cases of HFpEF, seric MPO concentrations were evaluated as potential early biomarkers for diastolic dysfunction. MPO concentrations were independently correlated with echocardiography parameters associated with diastolic dysfunction [146]. However, there are studies on animal subjects which failed to identify a positive correlation between MPO and HF severity [147].

### 2.11. Anti-Inflammatory Targeted Therapies in HF

Several trials have tried to evaluate the potential clinical effects of anti-inflammatory agents in heart failure, driven by the pathophysiological ability to counteract inflammatory cytokines. The trials are listed below, sorted by the targeted cytokine (Table 1).

Besides these trials of active substances, there are also other emerging therapies studied as potential treatments. One trial which is expected to have its conclusions this year is the REGRESS-HFpEF trial, which investigates the potential benefit of treatment with allogenic cardiosphere-derived cells delivered intracoronary in HFpEF patients [160]. The research is based on the role of mesenchymal stromal cells in the improvement of cardiac function [161]. Also, the mesenchymal stromal cells and their derived extracellular vesicles have shown a role in the modulation of T- and B-type lymphocytes [162], which resulted in an improvement in diastolic dysfunction and cardiac stiffening in a diabetes mellitus type 2 murine trial [163]. The REGRESS-HFpEF trial will focus on clinical functional status, myocardial interstitial fibrosis (evaluated using MRI), macroscopic fibrosis using delayed gadolinium enhancement and diastolic function [164].

Obesity leads to deposits of fat on vessels promoting not only atherosclerosis, but also visceral fat accumulation [165,166]. In normal conditions, the epicardium produces cytokines that contribute to the nourishment of the myocardium [167]. Because the epicardium has continuous contact with the myocardium through microcirculation, in chronic inflammatory diseases like metabolic syndrome, there is an overactivation of adipogenesis. This leads to the production of proinflammatory adipokines that can cause atrial and ventricular fibrosis, leading to HFpEF [167,168]. Besides bariatric surgery for the reduction of epicardial fat and systemic inflammation [169], there are also other pharmacological options, such as those used in the treatment of dyslipidaemia, diabetes mellitus and heart failure. For instance, statins (a dose of 80 mg of atorvastatin showed a reduction in the epicardial fat) [170] and antidiabetic drugs from the glucagon-like peptide 1 receptor agonist class (liraglutide and exenatide) reduced epicardial fat [171,172]. Blockade of the mineralocorticoid axis has also shown a reduction in the epicardial fat, by increasing the expression of adiponectin [173]. An inhibition of adipokines and proinflammatory cytokines is also cited in the context of neprilysin inhibitors, leading the way for future clinical trials of ARNI that could reduce the myocardial stiffening in HFpEF patients.

### 2.12. The Inflammatory Pathway of Microvascular Injury

Microvascular dysfunction, which appears in the context of atherosclerosis, surrounds cardiovascular disease, diabetes mellitus and chronic kidney disease [174]. In patients where atherosclerotic plaques are clearly identified, there is a strong recommendation for the prevention of cardiovascular events (myocardial infarction, stroke or acute peripheric ischemia) provided by antiplatelet therapy (with acetylsalicylic acid or clopidogrel) [175]. At least two clinical studies tried to find a better cardiovascular prevention strategy using an anticoagulant therapy in additionto antiaggregant therapy [176,177]. One of the studies (COMPASS) focused on the net clinical benefit of rivaroxaban 2.5 mg twice daily, added to acetylsalicylic acid 75 mg once daily [176]. This therapy showed a reduction in the cardiovascular events reaching statistical significance in a cohort of 27,935 high-risk patients with coronary or peripheral artery disease. And even more important is the fact that these results were also consistent in the high-risk subgroups and patients with multiple risk factors, while severe bleedings were less frequent and had smaller clinical impact [176]. This shows the clinical benefit in patients undertreated because of the fear of fatal or severe bleeding events, who can safely benefit from this combination [178]. However, other trials such as COMMANDER HF, evaluated the same therapy (rivaroxaban 2.5 mg twice daily versus placebo) in 5022 patients with coronary artery disease, hospitalized for worsening of chronic heart failure with LVEF of ≤40%. The results showed no superior protection against cardiovascular events (death, myocardial infarction or stroke) in the trial group versus placebo [177].

### 2.13. The role of Epigenetic Factors in Heart Failure

Beginning with the Framingham Heart Study, launched in 1948, which extended over more than 70 years, a correlation between biochemical, environmental, behavioural and genetic factors and cardiovascular disease has been observed [179]. More recent studies have shown that epigenetic modification has an important role in the pathophysiology of heart failure. Considered to be a major regulator mechanism of cell response to environmental change, epigenetic factors can induce a modulation of different gene functions, expressions or activities [180]. The main methods of epigenetic-induced cardiovascular disease are DNA methylation, histone modification and noncoding RNA regulation. 

DNA methylation, which is the most frequent form of gene expression regulation in mammals, transfers genetic information to offspring DNA, through DNA methyltransferases (DNMT) [181]. Madsen et al. showed in a study that the inhibition of DNMT3a function led to cardiomyocyte mitochondrial damage and impaired glucose metabolism [182]. In another study, Glezeva et al. found five genes (HEY2, MSR1, MYOM3, COX17 and miRNA-24-1) that were hypermethylated in the interventricular septum of patients with hypertrophic obstructive cardiomyopathy, ischemic cardiomyopathy and dilated cardiomyopathy [183]. Three other genes were in a hypomethylated state (CTGF, MMP2 and miRNA-155) [183]. Also, Zhu et al. showed that the inhibition of the DNMT2-induced DNA methylation of the glutathione peroxidase 1 gene promoter, through selenium supplementation, had a cardioprotective effect by reducing the production of reactive oxygen species inside cells and inhibiting cardiomyocyte apoptosis [184].

Histone modification can be produced by several processes such as: methylation, acetylation, phosphorylation, adenylation, ubiquitination and adenosine diphosphate ribosylation [185]. SIRT2 deficiency decreased AMPK activation, leading to age-related and angiotensin II-induced cardiac hypertrophy [186]. SIRT3 induces phosphorylation and degradation of SMAD3, reducing the TGF-beta induced myocardial fibrosis, while low levels of SIRT4 decreased angiotensin II-induced cardiac fibrosis [187]. In another study, Baldi et al. concluded that SIRT7 deacetylated p53, increasing the stress resistance and inhibiting myocardial apoptosis [188].

The noncoding RNAs are nucleotide structures that are not encoding proteins, consisting of ribosomal RNAs, transport RNAs, microRNAs (miRNAs), small interfering RNAs (siRNA), mRNA, small nuclear RNAs and small nucleolar RNAs [189]. Wu et al. showed that decreased levels of miRNA-92b-5p serum exosome in patients with acute heart failure were correlated with reduced ejection fraction [190]. In another trial Wang et al. observed that miRNA-425 and miRNA-744 inhibited angiotensin-induced collagen production, reducing the cardiac remodelling [191].

In conclusion, epigenetic factors can be regarded as future diagnostic markers and treatment targets in stopping heart failure progression [192].

## 3. Conclusions

Even if the components of the pathophysiological pathways responsible for cardiac fibrosis and remodelling, which further generate heart failure, have been studied for decades and in a major part already discovered, the importance of each of them is still yet to be completely established. The elevated levels of inflammatory cytokines and chemokines are present across the whole spectrum of chronic heart failure, irrespective of ejection fraction, and at the same time in acute heart failure and cardiogenic shock scenarios. 

The results of trials evaluating the effect of therapeutic agents targeting cytokines, aiming to inhibit the chronic low-grade inflammation present in heart failure, have proven to be unsuccessful until now. However, given the high prevalence of these inflammatory components in HFrEF and HFpEF, there is a great perspective that new clinical trials will succeed in identifying specific immunomodulators capable of reducing the mortality and morbidity of this cardiac burden. Due to the presence of diverse inflammatory infiltrate, with an established prognostic role, the treatment needs to be individualized and tailored accordingly, targeting the unique immunopathogenic profile corresponding to each particular individual.

## Figures and Tables

**Table 1:** The trials evaluating targeted therapies against pro-inflammatory cytokines. (↓ = decrease, ↑ = increase).

Target	Trial Acronym and Reference	Study Population	Sample Size	Intervention	Drug Mechanism of Action	Follow-Up	Outcomes
**TNF α**	**ATTACH** (Anti-TNF-α Therapy Against Congestive Heart Failure) Chung et al., 2003 [44]	HFrEF patients with III or IV NYHA functional class and LVEF ≤35%	150	Intervention group: 2 h intravenous infusion of **infliximab** 5 mg/kg (n = 50), 10 mg/kg (n = 51) at 0.2 and 6 weeks Placebo group (n = 49)	TNF-α inhibitor (anti-TNF mouse–human chimeric monoclonal antibody)	28 weeks	-No clinical status improvement at 14 weeks -10 mg/kg infliximab: ↑ death from any cause or hospitalization for HF -5 mg/kg: ↓ CRP, IL-6; LVEF ↑
	**RENEWAL** (Randomized Etanercept Worldwide Evaluation): combined data of RENAISSANCE and RECOVER trials in a prespecified study Mann et al., 2004 [17]	HFrEF patients with II to IV NYHA functional class and LVEF ≤ 30%	1673	Intervention group: **etanercept** subcutaneous injection 25 mg 3× weekly (n = 308), 25 mg 2× weekly (n = 683) Placebo group (n= 682)	TNF-α inhibitor (dimeric recombinant protein fusing the TNF receptor 2 to the Fc region of the human IgG1 antibody)	24 weeks	-No effect on the rate of death or hospitalization
**IL-1**	**CANTOS** (Canakinumab Anti-Inflammatory Thrombosis Outcome Study) Ridker et al., 2017 [148]	Patients with prior acute myocardial infarction and hsCRP ≥ 2 mg/L	10,061	**canakinumab** 50, 100 or 300 mg every 3 months	IL-1β inhibitor (monoclonal antibody blocking interaction between IL-1β and IL-1 receptors	48 months	-↓rate of recurrent cardiovascular events, independent of lipid level lowering
	**MRC-ILA** Morton et al., 2014 [149]	Acute NSTEMI (<48 h)	182	**anakinra** 100 mg daily	Inhibits IL-1 binding to the IL-1 type I receptor (recombinant, non-glycosylated form of the endogenous IL-1 receptor antagonist peptide)	2-week treatment (1 year follow-up)	-↓ CRP at 7 and 14 days (no effect on ischemic events at 30 days and 3 months, but ↑ at 1 year)
	**VCUART/VCUART 2/VCUART 3** Abbate et al., 2022 [150]	Acute STEMI (<12 h)	139	**anakinra, once or twice daily**	Inhibits IL-1 binding to the IL-1 type I receptor (recombinant, non-glycosylated form of the endogenous IL-1 receptor antagonist peptide)	2-week treatment (3 months and 1 year follow up)	-↓ CRP, ↓ incidence of HF, ↓ hospitalization for HF (no effect on ischemic events)
	**D-HART** (Diastolic Heart Failure Anakinra Response Trial) Van Tassell et al., 2014 [151]	HFpEF	12	**anakinra**, 100 subcutaneous daily for 28 days	Inhibits IL-1 binding to the IL-1 type I receptor (recombinant, non-glycosylated form of the endogenous IL-1 receptor antagonist peptide)	28 days	-↓ in CRP -↑ peak aerobic exercise capacity and quality of life at 2 weeks
	Van Tassell et al., 2016 [151]	Acute decompensated heart failure patients with LVEF ≤ 40%	30	**anakinra**, twice daily for 3 days, followed by once daily for 11 days	Inhibits IL-1 binding to the IL-1 type I receptor (recombinant, non-glycosylated form of the endogenous IL-1 receptor antagonist peptide)	14 days	-↓ systemic inflammatory response
	**REDHART** (Recently Decompensated Heart Failure Anakinra Response Trial) Van Tassell et al., 2017 [152]	Acute decompensated heart failure patients with LVEF ≤ 50%	60	**anakinra**, daily subcutaneous injection for 2 weeks, 12 weeks, or placebo	Inhibits IL-1 binding to the IL-1 type I receptor (recombinant, non-glycosylated form of the endogenous IL-1 receptor antagonist peptide)	24 weeks	-↓ CRP values -↑ peak VO_2_ (volume of oxygen consumption)
	**D-HART 2** (Diastolic Heart Failure Anakinra Response Trial 2) [151]	HFpEF patients	31	**anakinra**, 100 mg daily or placebo for 12 weeks	Inhibits IL-1 binding to the IL-1 type I receptor (recombinant, non-glycosylated form of the endogenous IL-1 receptor antagonist peptide)	24 weeks	-↓ CRP -↓ NT pro-BNP
	**AIR-HF** Van Tassell et al., 2012 [153]	Stable NYHA II-III HF patients with LVEF≤ 50% and CRP ≥ 2 mg/L	10	**anakinra**, single arm 200 mg daily for 14 days	Inhibits IL-1 binding to the IL-1 type I receptor (recombinant, non-glycosylated form of the endogenous IL-1 receptor antagonist peptide)	12 days	-↓ in CRP -↑ aerobic exercise capacity and ventilatory efficiency
**IL-6**	**RESCUE** (Reduction in Inflammation in Patients with Advanced Chronic Renal Disease Utilizing Antibody Mediated IL-6 Inhibition) Ridker et al., 2021 [154]	Moderate to severe patients with chronic kidney disease and hsCRP ≥ 2 mg/L, 9 high cardiovascular risk)	264	**ziltivekimab** 7.5 mg, 15 mg, 30 mg or placebo every 4 weeks	IL-6 antibody (monoclonal antibody directed against the IL-6 ligand)	24 weeks	-↓ hsCRP
**CRP**	**CORONA** (Controlled Rosuvastatin Multinational Trial in Heart Failure) Kjekshus et al., 2017 [155]	NYHA II to IV functional class HFrEF patients with LVEF ≤ 35%	5011	**rosuvastatin** 10 mg daily for at least 3 months or placebo	HMC-CoA reductase inhibitor with pleiotropic actions (antioxidant, anti-inflammatory, improvement of endothelial function)	32.8 months	-↓ CRP -↓ hospitalization for HF -No effect on the composite of cardiovascular-related death, non-fatal MI or stroke
	**GISSI-HF** (Gruppo Italiano Per Lo Studio Della Sopravvivenza Nell’Insufficienza Caridiaca-Heart Failure) Tavazzi et al., 2008 [156]	NYHA II to IV functional class ischemic and dilated cardiomyopathy with mean LVEF ≤ 45%	4574	**rosuvastatin** 10 mg daily for at least 3 months or placebo	HMC-CoA reductase inhibitor with pleiotropic actions (antioxidant, anti-inflammatory, improvement of endothelial function)	46.8 months	-↓ hsCRP values at 3 months -No effect on all-cause death or composite of all-cause death or hospitalization for cardiovascular causes
**NOS**	**LINCS** (L-NAME [a NO synthase inhibitor] in the treatment of refractory Cardiogenic Shock) Cotter et al., 2003 [157]	Refractory cardiogenic shock patients	30	Intervention group (n = 15): Supportive care in addition to **L-NAME**—1 mg/kg bolus 1 mg/kg/h continuous drip for 5 h; Control group (n = 15): supportive care alone	L-NAME (Non- selective NOS inhibitor)	4 months	-↑ blood pressure -↑ urinary output -↓ time of mechanical ventilation -↓ time of intra-aortic balloon pump support
	**SHOCK 2** (Should we inhibit NO in Cardiogenic Shock 2) Dzavik et al., 2007 [158]	Acute MI patients complicated by persistent cardiogenic shock despite PCI	79	Intervention groups (n = 15/15/15/14): **L-NMMA** 0.15/0.5/1/1.5 mg/kg/h infusion for 5 h; Placebo group (n = 20): 0.9% normal saline IV bolus	L-NAME (Non- selective NOS inhibitor)	2 h after study initiation (mean arterial pressure outcome) or 30 days (mortality outcome)	-↑ blood pressure at 15 min -No effect on blood pressure at 2 h -No effect on urinary output -No significant differences on mortality at 30 days
	**TRIUMPH** (Tilarginine Acetate Injection in a Randomized International Study in Unstable MI patients with Cardiogenic Shock) TRIUMPH Investigators et al., 2007 [159]	Acute MI patients complicated by persistent cardiogenic shock despite PCI	398	Intervention group (n = 206): **Tilarginine (LNMMA)**—1 mg/kg bolus and 1 mg/kg/h infusion for 5 h; Placebo group (n = 190)	L-NAME (Non- selective NOS inhibitor)	6 months	-No effect on 30-day all-cause mortality -↑ systolic blood pressure at 2 h -No effect on the resolution of shock, on reinfarction or on renal function
